# Rapid noncontact laser speckle imaging for perfusion-based differentiation of wound necrosis

**DOI:** 10.1016/j.isci.2026.117032

**Published:** 2026-08-06

**Authors:** Meng-Che Hsieh, Rou-Zheng Lai, Yan-Ren Lin, Ching-Han Hsu, Pei-You Hsieh, Wei-Fan Chen, Congo Tak Shing Ching, Lun-De Liao

**Affiliations:** 1 Institute of Biomedical Engineering and Nanomedicine, National Health Research Institutes, 35 Keyan Road, Zhunan Town, Miaoli County 350, Taiwan; 2 Doctoral Program in Tissue Engineering and Regenerative Medicine, National Chung Hsing University, 145 Xingda Road, South District, Taichung 402, Taiwan; 3 Department of Biomedical Engineering and Environmental Sciences, National Tsing Hua University, No. 101, Section 2 Kuang-Fu Road, Hsinchu 30013, Taiwan; 4 Department of Emergency Medicine, Chung Shan Medical University Hospital, Taichung 402, Taiwan; 5 School of Medicine, Chung Shan Medical University, Taichung 402, Taiwan; 6 Department of Emergency and Critical Care Medicine, Changhua Christian Hospital, Changhua, Taiwan; 7 Graduate Institute of Biomedical Engineering, National Chung Hsing University, 145 Xingda Road, South District, Taichung 402, Taiwan; 8 Department of Electrical Engineering, National Chi Nan University, Puli Township, Nantou County 54561, Taiwan; 9 International Doctoral Program in Agriculture, National Chung Hsing University, Taichung 402, Taiwan; 10 Advanced Plant and Food Crop Biotechnology Center, National Chung Hsing University, Taichung 402, Taiwan; 11 Institute of Intelligent Bioelectrical Engineering, National Yang Ming Chiao Tung University, Hsinchu City 300093, Taiwan; 12 Department of Biomedical Engineering, National Yang Ming Chiao Tung University, Taipei City 112304, Taiwan

**Keywords:** chronic wound, laser speckle contrast imaging, blood perfusion, wound healing prediction

## Abstract

Traditional wound assessment relies on subjective visual inspection, which may miss early microvascular changes. In this study, laser speckle contrast imaging (LSCI) was used to differentiate necrotic from nonnecrotic wounds using quantitative perfusion under a shortened protocol. Forty wound patients (20 acute, 15 chronic, and 5 necrotic) and 20 participants without wound lesions were included. Within-subject normalized perfusion (percent change relative to adjacent healthy skin) served as the predictor. Significant differences were observed among wound types (*ε*^2^ = 0.23–0.40, moderate-to-large effects). ROC analysis yielded an AUC of 0.88 (95% CI: 0.66–1.00; cutoff ≤−22.6%; accuracy 0.92; sensitivity 0.80; specificity 0.94; negative predictive value (NPV) 0.97). The 10-s protocol performed comparably to the 60-s acquisition (AUC = 0.84; accuracy = 0.87; *r* = 0.97; intraclass correlation coefficient (ICC) = 0.97). LSCI may offer a noncontact, rapid approach for perfusion-based necrosis differentiation, although the small necrotic sample (*n* = 5) necessitates validation in larger cohorts.

## Highlights

• LSCI differentiates necrotic from nonnecrotic wounds with 92% accuracy (AUC 0.88)
• LSCI shortens acquisition to 10 s with comparable diagnostic accuracy
• Noncontact LSCI evaluates wounds while reducing contamination risk
• LSCI reveals microvascular perfusion differences invisible on wound photographs

## Graphical abstract

**Figure:**
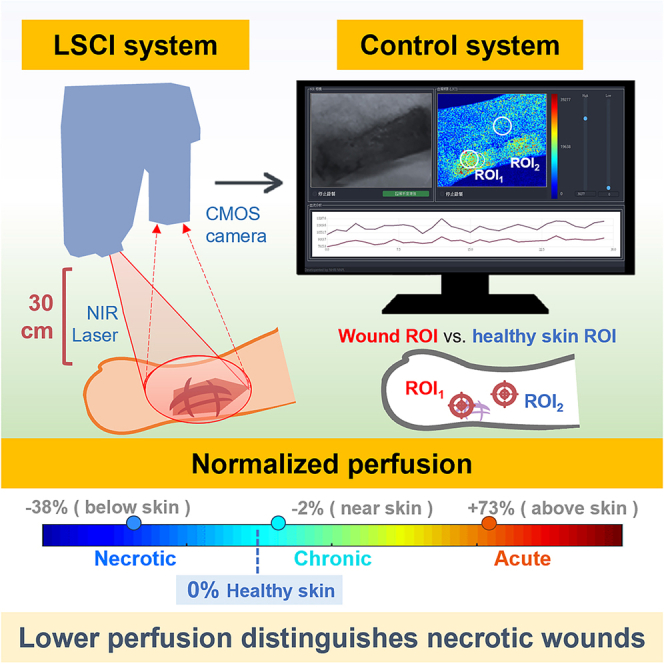

## Teaser

Optical imaging; Bioengineering

## Introduction

Wound assessment is a fundamental component of clinical care, with direct implications for treatment planning and patient outcomes. In clinical practice, wounds are generally classified as acute or chronic based on their etiology, healing trajectories, and duration. Chronic wounds, which are typically defined as wounds that fail to heal within a normal time frame and often persist for more than four weeks despite receiving appropriate treatment,^1^^,^^2^ commonly result from underlying conditions such as diabetes,^3^ vascular disorders,^4^ pressure injuries,^4^ or autoimmune diseases.^5^^,^^6^ These wounds often exhibit prolonged healing times and increased susceptibility to complications, including infections, and, if inadequately managed, may progress to tissue necrosis. In contrast, acute wounds arise from sudden external trauma, such as lacerations, burns, or surgical incisions.^1^ While acute wounds typically follow predictable healing phases when appropriately treated, inadequate or delayed interventions may result in complications ranging from infection to chronic ulceration and, in severe cases, tissue necrosis requiring surgical debridement or potentially amputation.

Traditional wound assessment procedures rely heavily on visual inspection and clinical experience,^7^ with healthcare professionals evaluating factors such as the size, depth, tissue type, and infection signs of wounds. While these methods remain essential to routine care, they are inherently subjective and depend heavily on clinician experience. Subtle changes in wound bed characteristics, particularly early microvascular alterations that may not be immediately apparent through visual examinations alone,^8^ are difficult to detect. This limitation is particularly consequential when attempting to identify wounds that are at risk for necrosis, where early interventions could significantly influence outcomes. An effective and objective assessment tool is therefore critical for providing timely and appropriate clinical interventions.^9^

To address these clinical assessment challenges, laser speckle contrast imaging (LSCI), a noncontact optical technique that enables real-time visualization and quantification of tissue perfusion, is employed in this study.^10^^,^^11^ When coherent laser light illuminates tissue, the resulting interference pattern (speckle) fluctuates in response to moving red blood cells.^12^ Regions with higher blood flow velocities exhibit more rapid speckle fluctuations and lower speckle contrast levels, whereas areas with reduced perfusion display more static patterns and higher contrast values.^10^ By computing speckle contrast values from acquired images, LSCI provides a two-dimensional map of the relative blood flow distribution across the examined tissue surface.^11^^,^^13^

LSCI performed using near-infrared wavelengths has an effective penetration depth of approximately 1 mm.^10^^,^^14^ Although this limits the investigation process to superficial tissue layers, it is sufficient for wound perfusion assessments because the superficial vascular plexus, which is located approximately 0.3–0.5 mm below the skin surface, represents the primary microvascular network supplying the epidermis and is directly relevant to wound healing and tissue viability evaluations.^15^^,^^16^ Clinical studies have validated the ability of LSCI to discriminate burn depths, predict wound healing outcomes, and assess tissue perfusion conditions in various types of wounds.^17^^,^^18^ This technique offers several practical advantages for clinical application: Its noncontact measurement process eliminates physical disturbances to healing tissue, its real-time imaging scheme enables the acquisition of immediate feedback, and its wide field of view allows for the simultaneous assessment of entire wound regions.^11^

However, several factors may influence the quality and interpretability of LSCI measurements. Surface characteristics, including skin pigmentation, the presence of crusts, scales, or wound exudate, and surface irregularities such as blisters, can affect optical signals and should be considered when positioning regions of interest (ROI) and interpreting perfusion values.^19^^,^^20^ Additionally, LSCI provides relative perfusion values expressed in arbitrary units that may vary between instruments depending on the configuration of the utilized system, making direct comparisons among the absolute values produced across different devices challenging to perform without system-specific calibration work.^10^^,^^21^

The application of LSCI technology has demonstrated significant versatility across various medical fields. LSCI has been applied for real-time cerebral blood flow imaging, demonstrating its ability to monitor continuous perfusion.^22^ Studies of burn assessment^17^ have shown that LSCI can determine burn depth and predict healing outcomes with high accuracy. In evaluations of diabetic foot ulcers,^23^ LSCI has proven valuable for assessing peripheral circulation and identifying areas that are at risk of ulceration before visible signs appear. In a previous study,^24^ we validated a custom LSCI system with an automatic region of interest tracking function that enabled consistent measurements to be acquired even when patients could not maintain completely stable positions, demonstrating high reliability, with intraclass correlation coefficients (ICCs) ranging from 0.798 to 0.917 for acute wounds and 0.628 to 0.849 for chronic wounds. However, the specific application of LSCI for distinguishing necrotic wounds from nonnecrotic wounds on the basis of quantitative perfusion thresholds remains incompletely characterized.

Building upon these technical foundations, this study aimed to evaluate the ability of LSCI to differentiate necrotic wounds from nonnecrotic wounds on the basis of quantitative perfusion measurements, establish preliminary perfusion cutoff values as clinical decision-support reference points, and validate the feasibility of a shortened 10-s imaging protocol for potential use in time-sensitive clinical environments. Early and objective identification of wound necrosis has particular clinical importance, as delayed recognition is associated with progressive tissue loss, systemic infection, and increased amputation risk; however, the existing assessment methods rely predominantly on visual inspections, which are inherently subjective and may fail to detect early microvascular compromises before macroscopic changes become apparent. Quantitative perfusion thresholds derived from LSCI measurements could therefore provide clinicians with an objective, noncontact adjunct for the early identification of necrosis. The clinical rationale for additionally evaluating a 10-s protocol reflects practical constraints that are inherent to bedside wound care: Patients with pain or limited mobility may be unable to remain still for extended durations, sequential multisite assessments require long acquisition periods, and wound exposure minimization reduces the risk of contamination. In this study, we therefore examined whether quantitative differences in perfusion exist among various wound types, with a particular focus on the contrast between necrotic and nonnecrotic wounds, and whether LSCI assessments can support the objective clinical discrimination process on this basis. The agreement between the 10-s and 60-s protocols was evaluated in parallel to determine the clinical feasibility of the shortened acquisition period.

## Results

**Figure 1:**
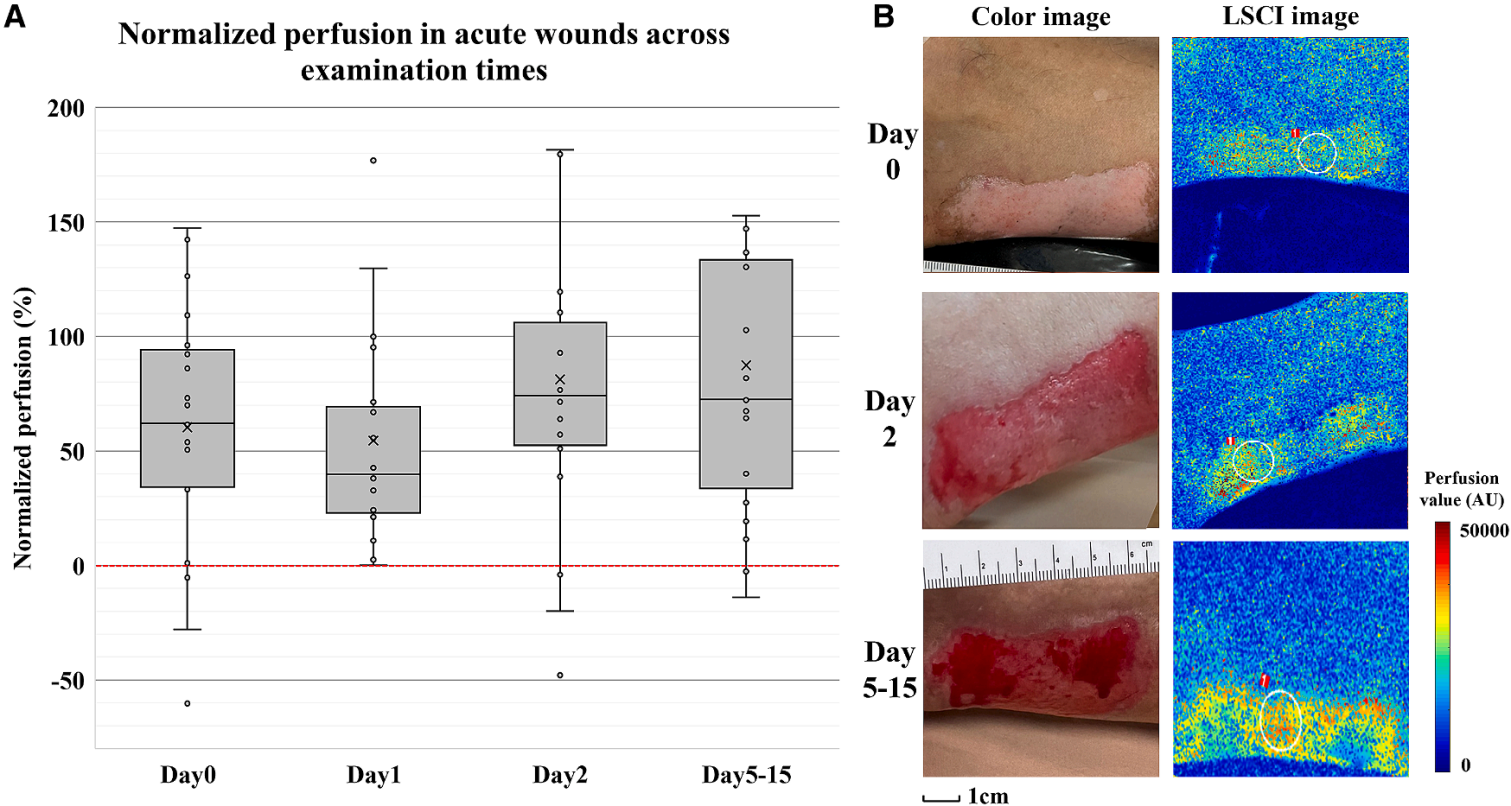
Normalized perfusion in acute wounds across different examination times (A) Boxplots of the within-subject normalized perfusion values acquired at four examination times (day 0, day 1, day 2, and days 5–15) following emergency department visits. Values above 0% (red line) indicate higher perfusion values in the wound tissue than in the adjacent healthy skin. Boxes show the interquartile range, center lines the median, and the cross marks the mean; whiskers extend to the most extreme values within 1.5× the interquartile range, and individual measurements are overlaid as open circles. (B) Representative color and LSCI images of acute wounds observed at different examination times, demonstrating the ability of LSCI to visualize perfusion differences that are not apparent in standard photographs. Scale bars, 1 cm.

The demographic, physiological, and clinical characteristics of the participants included in the chronic, necrotic, acute, and control groups (participants without wound lesions) are summarized in Tables 1 and 2. Table 1 summarizes the baseline characteristics and perfusion metrics of the chronic, necrotic, and control groups, whereas Tables 2 and 3 summarize the acute wound group and its time-specific normalized perfusion results. Table 1 additionally includes the absolute perfusion values of 20 participants without wound lesions as an external reference group; these participants had no wound sites and therefore no normalized values. Tables 2 and 3 report only the acute wound group, as the within-subject paired design inherently controlled for interindividual variability. The necrotic group had the greatest mean age (76.6 ± 21.4 years), followed by the chronic group (64.3 ± 15.1 years). As indicated in Table 1, the control group had a younger median age of 41.5 years, while the acute wound group (Table 2) had a median age of 46.0 years and a mean age of 47.3 ± 20.7 years. Physiological indicators such as blood pressure and oxygen saturation (SpO_2_) were generally lower in the necrotic group. Compared with the chronic group, the necrotic group had the lowest mean perfusion values, whereas the acute wound group generally exhibited higher absolute perfusion values across the follow-up measurements. Following the procedure described in the STAR Methods section, perfusion values in the wound groups were derived from wound-site ROIs, with simultaneously acquired adjacent healthy skin serving as the within-subject reference. In the control group (participants without wound lesions), perfusion values were obtained from intact skin ROIs. In the acute wound dataset, the normalized perfusion values obtained at the wound sites were consistently and significantly greater than those of the adjacent healthy skin throughout the observation period, as detailed in Table 3 and Figure 1. Although 20 patients with acute wounds were included, as shown in Table 3, ROI 3 data were missing for one patient (day 2) because of surgical debridement, and ROI 4 data were missing for another patient (days 5–15) because of a COVID-19 diagnosis, resulting in *N* = 19 participants for these respective time points. For cross-sectional comparisons involving the acute wound group, the fourth measurement (ROI 4, days 5–15) was used for analysis purposes. This time point corresponds to the proliferative phase of wound healing, when the initial inflammatory response has largely subsided, and microvascular perfusion becomes relatively stable. The use of ROI4 therefore reduces the influence of early inflammatory hyperemia and provides a more comparable physiological condition for chronic and necrotic wounds, which are typically assessed after the injuries have persisted for some time. With respect to their medical histories, the patients with chronic wounds had high incidence rates for hypertension and diabetes, whereas the patients with necrotic wounds presented more frequently with cardiovascular and cerebrovascular conditions. The patients with acute wounds demonstrated a moderate burden of comorbidities, including hypertension (25%), gastrointestinal diseases (30%), and cancer (15%). These baseline differences provide important context for interpreting the subsequent perfusion measurements and statistical comparisons.

**Table 1:** Baseline characteristics of the chronic wound, necrotic wound, and control groups

		Study group						Control group		
		Chronic group			Necrosis group			*N* = 20		
		*N* = 15			*N* = 5					
		N (%); Mean ± SD		Median	N (%); Mean ± SD		Median	N (%); Mean ± SD		Median
Gender	Male	9 (60)		–	3 (60)		–	7 (35)		–
	Female	6 (40)		–	2 (40)		–	13 (65)		–
Age (year)		64.3 ±	15.1	63.0	76.6 ±	21.4	81.0	42.4 ±	11.6	41.5
Height (cm)		162.1 ±	9.6	165.0	160.4 ±	15.4	155.0	162.7 ±	6.3	163.5
Weight (kg)		64.5 ±	13.0	63.0	61.9 ±	27.9	48.7	64.1 ±	10.5	61.0
BP (mmHg)	MAP	97.1 ±	16.5	98.3	92.3 ±	7.7	95.7	93.1 ±	13.9	91.7
	SBP	134.0 ±	23.4	138.0	135.0 ±	28.9	151.0	125.4 ±	21.1	124.0
	DBP	78.6 ±	17.2	81.0	71.0 ±	6.2	69.0	76.9 ±	11.6	78.0
BT (°C)		36.6 ±	0.9	36.1	36.6 ±	0.6	36.9	36.2 ±	0.1	36.2
HR (bpm)		87.1 ±	18.9	81.0	75.6 ±	5.2	77.0	76.2 ±	9.2	78.0
RR (breathing/min)		20.3 ±	1.4	20.0	20.0 ±	2.3	19.0	18.7 ±	0.9	18.0
SpO_2_ (%)		97.0 ±	3.6	98.0	94.4 ±	2.6	94.0	99.1 ±	0.4	99.0
Perfusion value (AU)		26,804 ±	11,563	21,557	12,710 ±	8,257	13,574	17,601 ±	5,388	16,684
Normalized perfusion value (%)		13 ±	53	−2	−34 ±	45	−38	–		–
Medical history	H.T.N.	8 (53)			2 (40)			1 (5)		
	D.M.	8 (53)			2 (40)			2 (10)		
	H.L.D.	4 (27)			1 (20)			0 (0)		
	C.V.D.	1 (7)			3 (60)			0 (0)		
	L.C.	1 (7)			1 (20)			0 (0)		
	C.V.A.	2 (13)			1 (20)			0 (0)		
	R.F.	5 (33)			2 (40)			0 (0)		
	GI diseases	6 (40)			2 (40)			1 (5)		
	I.T.P.	1 (7)			1 (20)			0 (0)		
	C.A.	7 (47)			0 (0)			5 (25)		

Data are n (%) or mean ± SD; median is shown separately. Normalized perfusion (%) = % change vs. adjacent healthy skin.

MAP, mean arterial pressure; SBP/DBP, systolic/diastolic blood pressure; BT, body temperature; HR, heart rate; RR, respiratory rate; SpO_2_, peripheral oxygen saturation; AU, arbitrary units; H.T.N., hypertension; D.M., diabetes mellitus; H.L.D., hyperlipidemia; C.V.D., cardiovascular disease; L.C., liver cirrhosis; C.V.A., cerebrovascular accident; R.F., renal failure; GI, gastrointestinal; I.T.P., immune thrombocytopenia; C.A., cancer.

**Table 2:** Baseline characteristics of the acute wound group

		Acute group (*N* = 20)		
		n (%); Mean ± SD		Median
Gender	Male	11 (55)		–
	Female	9 (45)		–
Age (year)		47.3 ±	20.7	46.0
Height (cm)		165.7 ±	9.1	164.5
Weight (kg)		69.4 ±	18.2	64.5
BP (mmHg)	MAP	101.9 ±	13.9	101.9
	SBP	143.8 ±	17.8	140.0
	DBP	84.1 ±	14.6	80.5
BT (°C)		36.4 ±	0.5	36.5
HR (bpm)		86.9 ±	19.5	89.0
RR (breathing/min)		21.2 ±	6.8	20.0
SpO_2_ (%)		99.3 ±	0.7	99.0
Medical history	H.T.N.	5 (25)		
	D.M.	2 (10)		
	H.L.D.	2 (10)		
	C.V.D.	1 (5)		
	L.C.	0 (0)		
	C.V.A.	0 (0)		
	R.F.	1 (5)		
	GI diseases	6 (30)		
	I.T.P.	0 (0)		
	C.A.	3 (15)		

Data are n (%) or mean ± SD; medians. AU, arbitrary units.

MAP, mean arterial pressure; SBP/DBP, systolic/diastolic blood pressure; BT, body temperature; HR, heart rate; RR, respiratory rate; SpO_2_, peripheral oxygen saturation; H.T.N., hypertension; D.M., diabetes mellitus; H.L.D., hyperlipidemia; C.V.D., cardiovascular disease; L.C., liver cirrhosis; C.V.A., cerebrovascular accident; R.F., renal failure; GI, gastrointestinal; I.T.P., immune thrombocytopenia; C.A., cancer.

**Table 3:** Normalized perfusion in the acute wound group across examination times

Examination time	Perfusion value (AU)		Normalized perfusion (%)		*p* value	Effect size (*r*)
	Mean ± SD	Median	Mean ± SD	Median		
ROI 1 (Day 0)	30,850 ± 17,301	27,102	57 ± 56	62	<0.05	0.73
ROI 2 (Day 1)	30,598 ± 12,888	28,142	56 ± 44	41	<0.05	0.88
ROI 3^a^ (Day 2)	32,125 ± 11,854	32,154	85 ± 74	77	<0.05	0.81
ROI 4^b^ (Day 5–15)	36,809 ± 14,765	34,665	87 ± 75	73	<0.05	0.84

Data are mean ± SD, median. Perfusion values are in arbitrary units (AU). Normalized perfusion (%) = percentage change relative to adjacent healthy skin. The *p* values and effect sizes (*r*) refer to the comparison of normalized perfusion against zero (adjacent healthy skin) at each examination.

a ROI 3: *N* = 19 (Data missing for one patient due to surgical debridement and the placement of artificial dermis at the plastic surgery outpatient clinic).

b ROI 4: *N* = 19 (Data missing for one patient due to a COVID-19 diagnosis requiring home isolation and self-wound care, preventing the scheduled follow-up.).

### Differences between wounded skin and the adjacent healthy skin

The within-subject normalized perfusion changes exhibited by the patients with acute wounds at four examination time points, namely, day 0, day 1, day 2, and days 5–15, are shown in Figure 1A. The perfusion values were normalized using the adjacent healthy skin as a reference and expressed as percentage changes, thereby reducing interindividual variability and enabling comparisons among the relative perfusion differences observed over time. Table 3 summarizes the corresponding normalized perfusion values obtained for the wound sites relative to the adjacent healthy skin. The median normalized perfusion changes were 62% with an interquartile range of 25%–93% on day 0, 41% with an interquartile range of 23%–74% on day 1, 77% with an interquartile range of 54%–115% on day 2, and 73% with an interquartile range of 34%–134% on days 5–15. Wilcoxon signed-rank tests indicated that the normalized perfusion values were significantly greater than 0% at all examination time points (all *p* < 0.05), with large effect sizes (*r* = 0.73–0.88), indicating substantial differences between the perfusion values of wounds and the adjacent healthy skin. One patient missed the day-2 assessment because of debridement and the placement of artificial dermis at the plastic surgery outpatient clinic. Another participant was unable to attend the day-5–15 follow-up visits because of a COVID-19 diagnosis. These absences resulted in minor sample size variations across the different time points. Overall, the findings demonstrate that perfusion was persistently elevated in acute wounds relative to the adjacent healthy skin throughout the early healing period.

### Multivariable regression analysis adjusted for covariates

To further examine whether the differences in perfusion were attributable to the type of wound rather than baseline characteristics, a multivariable linear regression analysis was conducted. In the fully adjusted model, which included age, mean arterial pressure, and diabetes status, the wound type remained independently associated with perfusion after adjustment. Compared with the acute wound group (reference), the necrotic group demonstrated significantly lower perfusion values (*β* = −19,910 AU; *p* < 0.05), and the control group also had significantly lower perfusion values (*β* = −14,280 AU; *p* < 0.05). The chronic wound group did not significantly differ from the acute wound group after the adjustment occurred (*p* > 0.05). Age (*p* > 0.05), MAP (*p* > 0.05), and diabetes status (*p* > 0.05) were not independently associated with perfusion in the adjusted model. A variance inflation factor analysis confirmed the absence of significant multicollinearity among the predictors (all VIFs < 2).

### Differences among the LSCI results obtained for acute, chronic, and necrotic wounds

**Figure 2:**
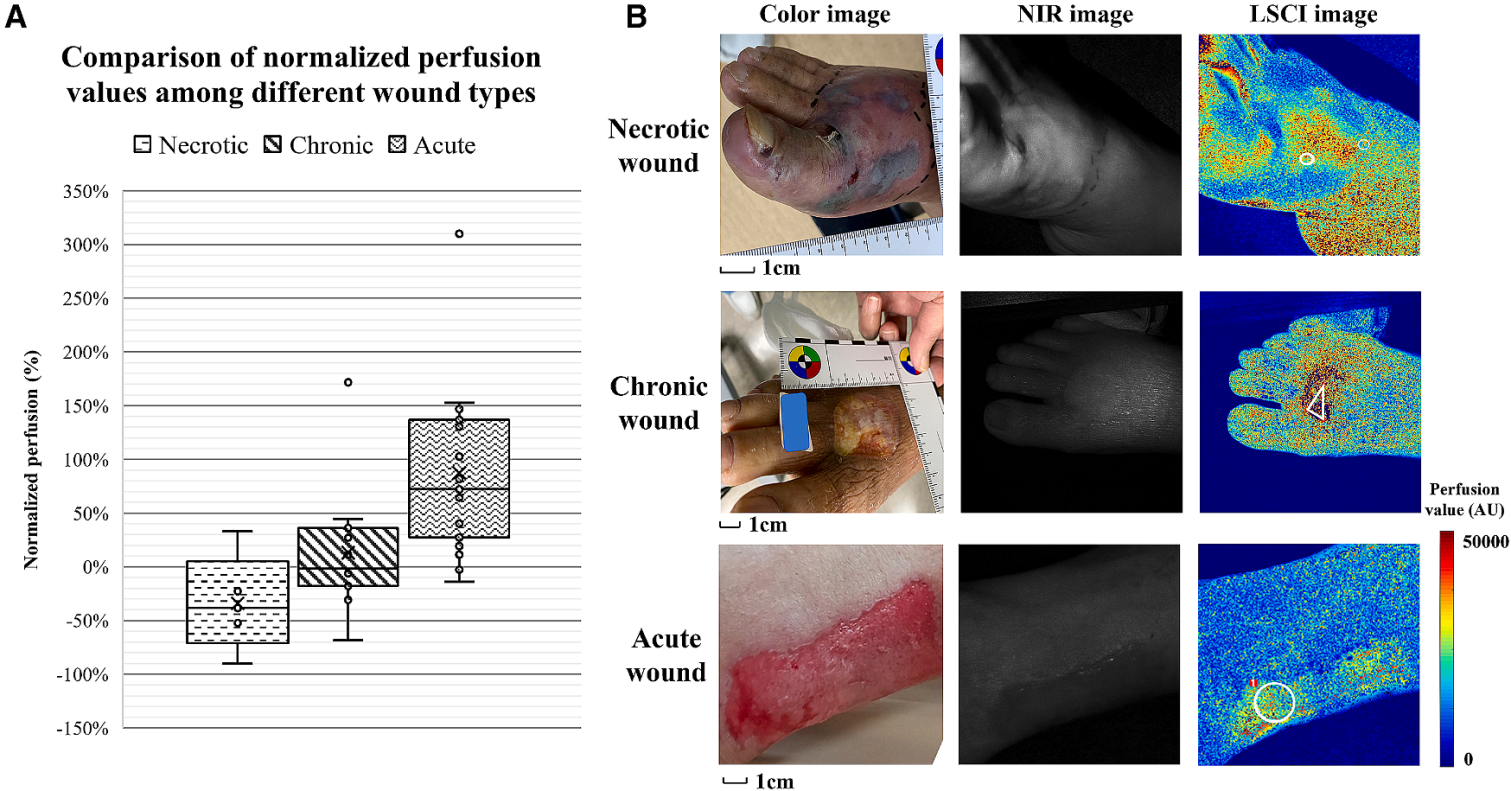
Comparisons among the normalized perfusion values obtained for different wound types (A) Boxplots showing the normalized perfusion values produced for necrotic, chronic, and acute wounds. The perfusion values were normalized relative to those of the adjacent healthy skin. Necrotic wounds presented the lowest normalized perfusion values, whereas chronic and acute wounds presented relatively high perfusion levels. Boxes show the interquartile range, center lines the median, and the cross marks the mean; whiskers extend to the most extreme values within 1.5× the interquartile range, and individual measurements are overlaid as open circles. (B) Representative color, NIR, and LSCI images of acute, chronic, and necrotic wounds. While color images effectively document surface wound characteristics, LSCI provides complementary quantitative perfusion data that reflect underlying microvascular statuses. The color scale represents the relative perfusion values, with warmer colors indicating higher blood flow. Scale bars, 1 cm.

A boxplot of the normalized perfusion values obtained for the different wound types is shown in Figure 2A, and clear differences in perfusion levels are observed. Necrotic wounds tended to have the lowest normalized perfusion values, acute wounds had the highest values, and chronic ulcers had an intermediate distribution. Color, NIR, and LSCI images of different wound types are shown in Figure 2B. While the color and NIR images provided mainly structural information, the LSCI images revealed distinct perfusion patterns that enabled clearer differentiation among acute, chronic, and necrotic wounds. As shown in Table 4, the necrotic wounds had significantly lower perfusion values than the chronic ulcers (*p* < 0.05), with an effect size of *r* = 0.26, indicating a small-to-moderate effect. The results of the Kruskal–Wallis test performed for the necrotic wounds, chronic ulcers, and control group revealed statistically significant differences (*p* < 0.05), with an effect size of *ε*^2^ = 0.23. Post hoc pairwise comparisons conducted using Dunn’s test with Holm correction revealed significant differences between the necrotic and chronic wounds (*p* < 0.05 after Holm correction) and between the chronic ulcers and the control group (*p* < 0.05 after Holm correction). The table also compares the necrotic wounds, chronic ulcers, and acute wounds measured during the fourth examination (days 5–15). The results of the Kruskal–Wallis test again demonstrated significant differences among the groups (*p* < 0.05), with an effect size of *ε*^2^ = 0.30. Dunn’s post hoc analysis with Holm adjustment revealed a significant difference between the necrotic and acute wounds (*p* < 0.05 after Holm correction), whereas the other pairwise comparisons were not statistically significant after the correction was applied. When the perfusion values were normalized relative to those of the adjacent healthy skin, the results of the Kruskal–Wallis test also revealed significant differences among the three wound types (*p* < 0.05, *ε*^2^ = 0.40). Dunn’s post hoc analysis further revealed that normalized perfusion was significantly greater in acute wounds than in both necrotic and chronic wounds.

**Table 4:** Comparisons of perfusion values among wound types

Analysis	Group (n)	Mean (SD)	Median	*p* value	Effect size	Post hoc (Dunn–Holm)
Necrotic vs. chronic perfusion (AU); Mann–Whitney U	Necrosis (*n* = 5)	12,710 (8,257)	13,574	<0.05	*r* = 0.26	–
	Chronic ulcer (*n* = 15)	26,804 (11,563)	21,557			
Necrotic, chronic, control perfusion (AU); Kruskal–Wallis	Necrosis (*n* = 5)	12,710 (8,257)	13,574	<0.05	*ε*^2^ = 0.23	N–C (<0.05); C–Ctrl (<0.05)
	Chronic ulcer (*n* = 15)	26,804 (11,563)	21,557			
	Control (*n* = 20)	17,601 (5,388)	16,684			
Necrotic, chronic, acute 4th exam perfusion (AU); Kruskal–Wallis	Necrosis (*n* = 5)	12,710 (8,257)	13,574	<0.05	*ε*^2^ = 0.30	N–A (<0.05)
	Chronic ulcer (*n* = 15)	26,804 (11,563)	21,557			
	Acute, 4th exam (*n* = 19)	36,809 (14,765)	34,665			
Necrotic, chronic, acute 4th exam normalized perfusion (%); Kruskal–Wallis	Necrosis (*n* = 5)	−34 (45)	−38	<0.05	*ε*^2^ = 0.40	N–A (<0.05); C–A (<0.05)
	Chronic ulcer (*n* = 15)	13 (53)	−2			
	Acute, 4th exam (*n* = 19)	87 (75)	73			

The Mann–Whitney U test was used for the two-group comparison, and the Kruskal–Wallis test with Dunn’s post hoc test (Holm correction) for the three-group comparisons. Effect sizes: *r* (Mann–Whitney U) and *ε*^2^ (Kruskal–Wallis). Perfusion values are in arbitrary units (AU); normalized perfusion (%) = percentage change relative to adjacent healthy skin.

N, necrotic wounds; C, chronic ulcers; A, acute wounds; Ctrl, control group. Statistical significance was defined as *p* < 0.05.

### ROC curve-based discrimination of necrotic vs. nonnecrotic wounds

**Figure 3:**
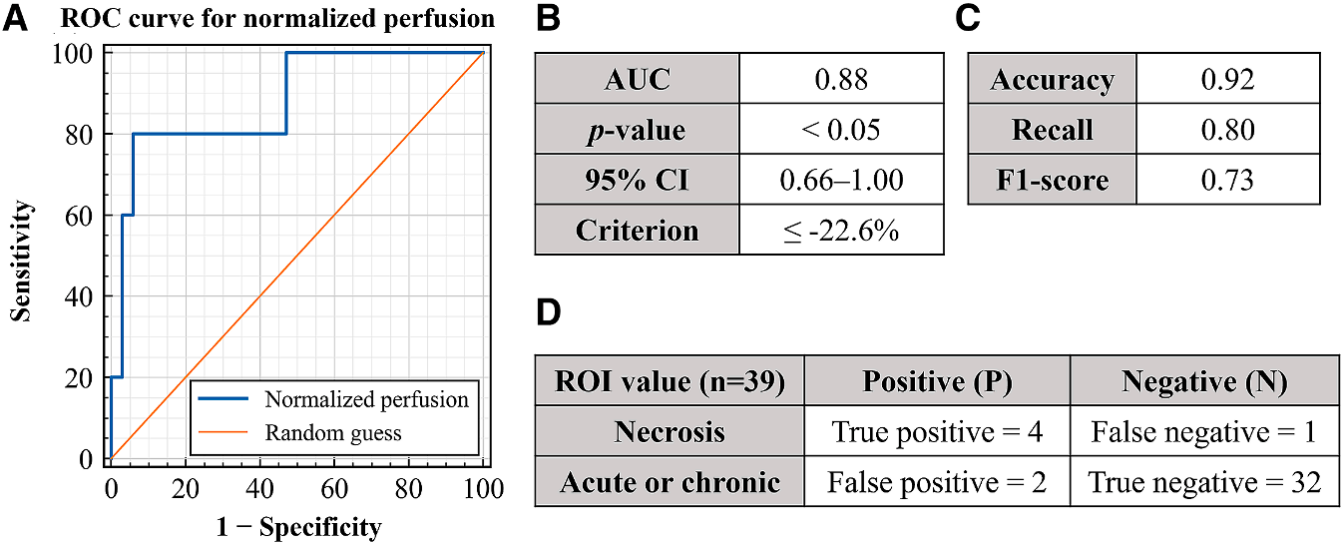
ROC analysis of the normalized perfusion values employed for differentiating necrotic wounds (A) ROC curve produced on the basis of the within-subject normalized perfusion values, expressed as percentage differences relative to the adjacent healthy skin. (B) Statistical significance and AUC metrics. (C) Classification performance metrics calculated at the selected cutoff threshold. (D) Confusion matrix produced for nonnecrotic (acute or chronic) and necrotic wounds (*n* = 39). Positive indicates necrotic wounds.

To evaluate the ability of LSCI to differentiate necrotic wounds from nonnecrotic wounds, an ROC curve analysis was performed using the ROI measurements derived from 39 patients (19 acute wounds, 15 chronic ulcers, and 5 necrotic wounds). The predictor variable was the within-subject normalized perfusion value, expressed as the percentage difference relative to the adjacent healthy skin. As shown in Figures 3A and 3B, the ROC curve analysis yielded an AUC of 0.88 (95% CI: 0.66–1.00), indicating good discriminatory ability in differentiating necrotic wounds from nonnecrotic wounds. The optimal cutoff was determined to be ≤ −22.6%, indicating that necrosis was more likely when the normalized perfusion value was ≤ −22.6% relative to the adjacent healthy skin. For classification analysis, “positive (P)” was defined as a necrotic wound, whereas “negative (N)” represented a nonnecrotic wound (acute or chronic). Among the 5 necrotic wounds, 4 were correctly identified as positive (true positives), and 1 was misclassified as nonnecrotic (false negative). Among the 34 nonnecrotic wounds, 32 were correctly classified as negative (true negatives), and 2 were misclassified as necrotic (false positives). As shown in Figures 3C and 3D, these results corresponded to an overall accuracy of 0.92, a sensitivity of 0.80, a specificity of 0.94, and an F1 score of 0.73 for detecting necrosis. Bootstrap-derived 95% confidence intervals were estimated for all the classification metrics, including sensitivity, specificity, positive predictive value (PPV), negative predictive value (NPV), and the optimal cutoff. The PPV was 0.67 (95% CI: 0.12–1.00), whereas the NPV was 0.97 (95% CI: 0.91–1.00), indicating a strong ability to exclude necrosis when the perfusion reduction did not meet the cutoff threshold. The resulting confidence intervals for sensitivity (0.60–1.00), specificity (0.49–1.00), PPV (0.12–1.00), NPV (0.91–1.00), and the optimal cutoff (−52.2%–33.0%) are reported to provide an estimate of the variability around the point estimates. Given the small necrotic sample size (*n* = 5), the bootstrap-derived confidence intervals were wide and should be interpreted with caution.

### Wound healing trends

As shown in Figure 1A, boxplots of the within-subject normalized perfusion changes were analyzed from day 0 to day 15 in the acute wound group. The normalized perfusion values were consistently and significantly elevated above the adjacent healthy skin baseline throughout the observation period. This trend is quantitatively supported by the normalized perfusion data presented in Table 3. At all four time points, the normalized perfusion values were significantly greater than 0% (all *p* < 0.05), with large effect sizes (*r* = 0.73–0.88).

No consistent temporal trend in the group-level normalized perfusion was observed across the follow-up period. Despite interindividual variability, the wound type remained independently associated with perfusion after adjusting for age, mean arterial pressure, and diabetes status, confirming that the observed differences are not solely attributable to baseline demographic or comorbidity variations.

### Results of the imaging time comparison

**Figure 4:**
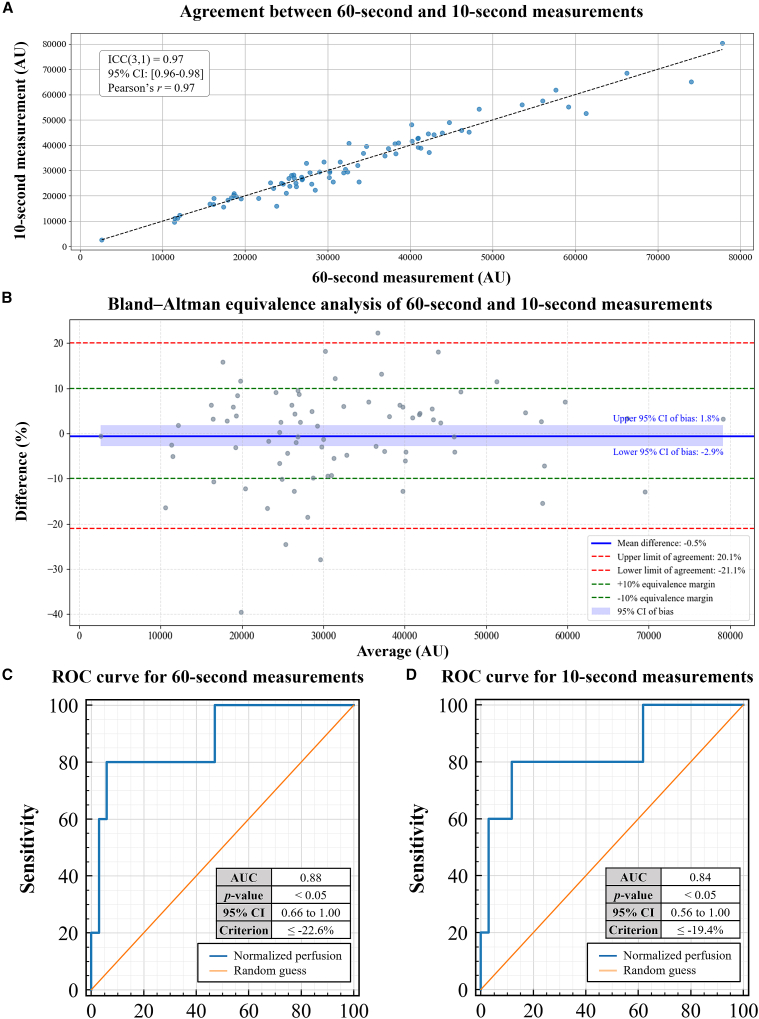
Comparison between the results of the 60-s and 10-s imaging measurement durations The dataset consists of 78 ROI perfusion measurements acquired from acute wound patients. (A) Scatterplot compares the perfusion values between the two durations, with correlation statistics. (B) Bland-Altman plot showing the agreement between the measurements. (C) ROC curve analysis of the 60-s measurements for differentiating necrotic wounds. (D) ROC curve analysis of the 10-s measurements, demonstrating comparable diagnostic performance.

We evaluated the level of agreement between the 60- and 10-s LSCI recordings on the basis of a total of 78 paired ROI measurements derived from acute wounds (Table 5) to assess whether a shorter acquisition time could yield comparable perfusion measurements. The 10-s data were extracted from the first 10 s of each corresponding 60-s recording. The relationship between the perfusion values obtained at the two acquisition durations is shown in the scatterplot in Figure 4A. Visual inspection of the scatterplot indicates strong agreement between the two datasets. Quantitatively, the Pearson’s correlation coefficient was 0.97, and the ICC (3, 1) was also 0.97, with a 95% CI ranging from 0.96 to 0.98, indicating excellent consistency between the full and shortened recordings. The degree of agreement was further evaluated using the Bland–Altman analysis method, as shown in Figure 4B. The mean bias between the 60- and 10-s perfusion values was −0.5%. The mean bias was small, and its 95% CI ranged from −2.9% to 1.8%, remaining entirely within the predefined equivalence margin of ±10%, although the 95% limits of agreement were wider (−21.1%–20.1%). These findings indicate strong overall agreement with limited systematic bias but also suggest that individual paired differences may still be substantial in some cases. To further examine whether the shortened recording duration affected the diagnostic performance, an ROC analysis was conducted using the normalized perfusion values derived from 39 patients, including 19 with acute wounds, 15 with chronic ulcers, and 5 with necrotic wounds. The 60-s dataset yielded an AUC of 0.88 (95% CI: 0.66–1.00; *p* < 0.05), with an overall classification accuracy of 0.92 and an optimal cutoff of ≤ −22.6%, as shown in Figure 4C. As shown in Figure 4D, the 10-s measurements yielded an AUC of 0.84 (95% CI: 0.56–1.00; *p* < 0.05), with an accuracy of 0.87 and an optimal cutoff of ≤ −19.4%. Although the 10-s protocol yielded a modest AUC reduction, the bootstrap confidence intervals overlapped between the two measurements, and both remained statistically significant. These findings suggest that the shortened acquisition duration maintains clinically meaningful discriminatory performance, but we acknowledge the uncertainty associated with the limited number of necrotic cases in this cohort.

**Table 5:** Sample size and statistical methods used across the experiment

Statistical method	Necrosis	Chronic	Acute	Control group
Mann‒Whitney U test (Table 4 Necrotic vs. chronic)	5	15	–	–
Kruskal‒Wallis test (Table 4 Necrotic, chronic, control)	5	15	–	20
Kruskal‒Wallis test^a^ (Table 4 Necrotic, chronic, acute 4th exam)	5	15	19 (4th examination)	–
ROC curve analysis^a^ (Figure 3)	5	15	19 (4th examination)	–
ICC and Pearson correlation analyses (Figure 4A)	–	–	78 measurements (from 20 patients with acute wounds)	–
Bland–Altman analysis (Figure 4B)	–	–	78 measurements (from 20 patients with acute wounds)	–
ROC curve analysis^a^ (Figures 4C and 4D)	5	15	19 (4th examination)	–

ROC, receiver operating characteristic; ICC, intraclass correlation coefficient.

a *N* = 19 (data were missing for one patient due to a COVID-19 diagnosis, so no follow-up was performed).

## Discussion

### Wound healing trends and physiological interpretation

The consistently elevated normalized perfusion values observed in acute wounds relative to those in adjacent healthy skin throughout the early observation period (Table 3) reflect the physiological wound healing process.^1^ During the first two weeks of healing, wound recovery involves several overlapping physiological phases.^1^^,^^4^ The initial inflammatory response triggers increased blood flow to support immune cell infiltration and debris removal,^4^^,^^5^ as reflected by our LSCI data showing significantly elevated perfusion values from day 0 onward.

**Figure 5:**
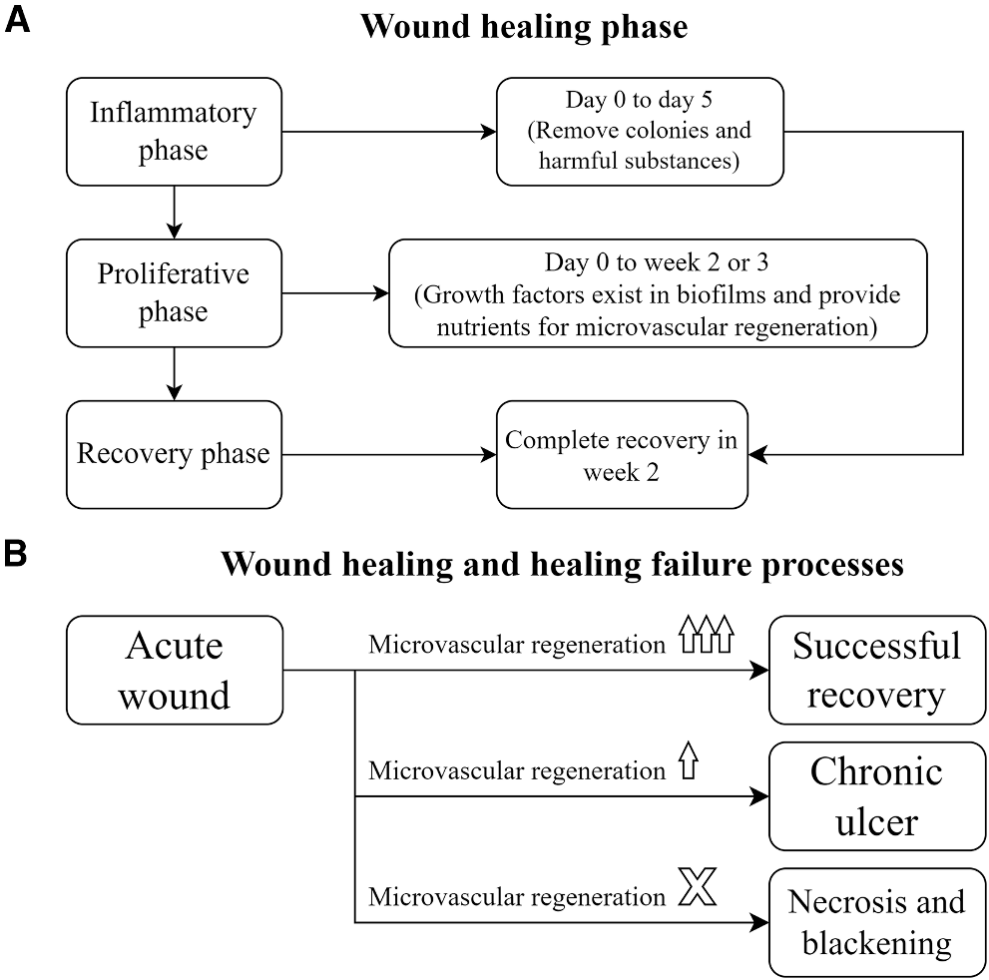
Physiological stages of wound healing and the process of healing failure (A) The three overlapping stages of normal wound healing: inflammation, proliferation, and remodeling. (B) Microscopic processes associated with wound healing and healing failure. Successful healing requires the proliferation and neovascularization of epidermal cells to supply nutrients for the formation of granulation tissue. Neovascularization failure can result in chronic nonhealing wounds.

These physiological phases are schematically illustrated in Figure 5A, which depicts the coordinated sequence of inflammation, proliferation, and remodeling during the acute wound healing process. Day 0 corresponds to the transition from hemostasis to early inflammation, when vasodilation and increased vascular permeability facilitate immune cell infiltration.^4^^,^^6^ Days 1–2 represent the inflammatory peak, during which neutrophils and macrophages accumulate at the wound site to clear necrotic debris; the sustained high perfusion during this phase reflects active metabolic demands and may assist in early perfusion-based assessment of wound status.^5^^,^^6^ Days 5–15 encompass the transition to the proliferative phase, which is characterized by angiogenesis and granulation tissue formation.^1^^,^^4^ Our LSCI measurements captured these microvascular changes, with elevated perfusion values reflecting active neovascularization trends during this period.

In contrast, the disrupted healing cascade in the chronic wound group, in which persistent inflammation and impaired angiogenesis led to delayed tissue regeneration, is shown in Figure 5B.^5^^,^^25^ The significantly different perfusion profiles observed for acute and chronic wounds in Table 4 align with these distinct pathophysiological trajectories.

The absence of a clear temporal trend in our data can be attributed to the heterogeneity exhibited by the wound characteristics. Variations in wound depth, size, etiology, and anatomical location affect healing trajectories, as deep wounds involving multiple tissue layers typically require longer healing times than superficial injuries do.^1^^,^^4^^,^^5^ Nevertheless, the statistically significant increase in perfusion observed at each time point suggests that LSCI provides clinically meaningful information when applied to intraindividual comparisons, even in the absence of a global trend. Future studies should stratify wounds according to these characteristics to better characterize the temporal changes in perfusion observed during the healing process.

### Necrosis discrimination and indicators

**Figure 6:**
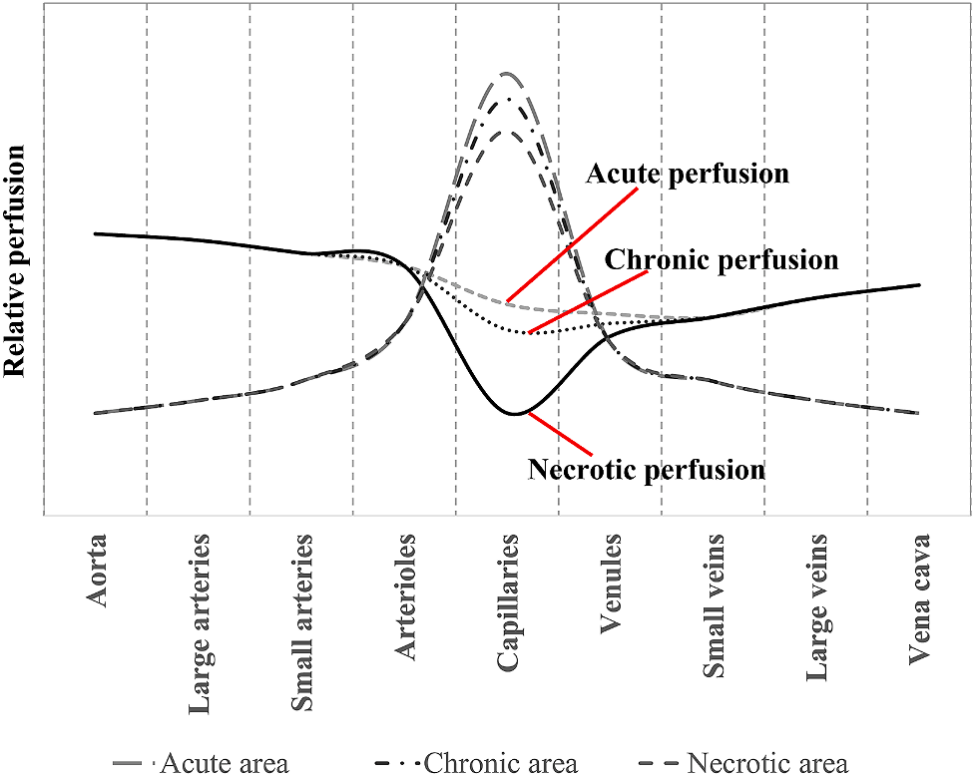
Illustration of microvascular perfusion and vascular area Schematic representation of microvascular perfusion distributions along the vascular network in acute, chronic, and necrotic wounds. The diagram illustrates the differences in relative angiogenesis and microvascular perfusion among the three wound types.

We further analyzed the normalized perfusion values of different types of wounds by constructing boxplots to assess their wound status. The box plot of the perfusion values for different wound types in Figure 2A shows a separation trend, with necrotic wounds tending to cluster at lower normalized perfusion values than chronic and acute wounds did, although partial overlap remained across groups. The color, NIR, and LSCI images of different wound types clearly differ on the basis of microvascular perfusion patterns and are shown in Figure 2B. A schematic representation of the microvascular perfusion distribution and vascular area along the vascular network is shown in Figure 6.^26^ Necrotic wounds exhibit severely reduced microvascular perfusion because of impaired angiogenesis and microcirculatory collapse, resulting in markedly lower perfusion values. Chronic ulcers,^25^ although characterized by impaired or incomplete microvascular neogenesis, still maintain partial microvascular circulation, leading to intermediate perfusion levels relative to those of necrotic and acute wounds. In contrast, acute wounds typically demonstrate active angiogenesis and increased microvascular perfusion, resulting in higher perfusion values and greater healing potential. Compared with the chronic wound group, the control group, which consisted of participants without wound lesions, presented lower absolute perfusion values; this finding is consistent with the finding that the resting perfusion of intact skin in the absence of wound-related inflammatory angiogenesis is distinct from the pathological vascular remodeling depicted in Figure 6. As detailed in Table 4, nonparametric analyses confirmed significant differences in perfusion among the wound types, with necrotic wounds exhibiting the lowest values. The most pronounced contrast in perfusion was observed between the wounds clinically diagnosed as necrotic and higher-perfusion acute wounds, whereas the intermediate wound types showed partial overlap in perfusion distributions. Effect size analyses demonstrated moderate-to-large effects (*ε*^2^ = 0.23–0.40) across all wound type comparisons, indicating that the wound type explained a considerable proportion of the variance in perfusion values. These effect sizes support the clinical relevance of LSCI-based wound discrimination and suggest that the differences in perfusion between various wound types are not only statistically significant but also clinically meaningful.

On the basis of these test results, we identified a preliminary threshold for differentiating necrotic wounds from nonnecrotic wounds. This threshold may serve as an initial reference for assessing wound perfusion status, although validation in larger cohorts is needed before clinical implementation. An ROC curve analysis^27^ was performed to identify an optimal cutoff for differentiating necrotic wounds from nonnecrotic wounds. When the Youden index was used, the optimal cutoff for the within-subject normalized perfusion change was ≤ −22.6%. Therefore, wounds with normalized perfusion changes ≤ −22.6% were classified as necrotic, whereas those with normalized perfusion changes > −22.6% were classified as nonnecrotic. Such assessments may inform individualized wound treatment decisions. By determining the differences in perfusion between different wound types, medical professionals can better understand the healing processes of wounds and develop more effective treatment strategies for different situations.

These findings provide preliminary evidence supporting perfusion-based wound assessment. By providing early perfusion data within the first 3–5 days after injury, LSCI enables clinicians to identify impaired healing potential earlier, reduce unnecessary dressing changes, and avoid secondary injuries caused by repeated tissue exposures. Compared with the traditional visual inspection scheme alone, LSCI offers quantitative guidance for providing earlier interventions when needed. Notably, in this study, LSCI measurements in the necrotic group were performed after clinical debridement of the eschar and devitalized surface tissue. This protocol was designed to expose the underlying wound bed and avoid optical attenuation by nonperfused surface material, thereby enabling the assessment of residual perfusion capacity within wounds clinically diagnosed as necrotic. The reduced perfusion observed in these wounds is consistent with regional microvascular compromise, which, in conditions such as diabetic foot disease or peripheral vascular insufficiency, reflects a chronic pathophysiological process rather than an acute hemodynamic event. Such microvascular impairment persists in the tissue beneath and surrounding the necrotic area regardless of whether surface debridement has been performed. Although debridement may induce transient hyperemia at the wound margin, the within-subject normalization approach used in this study captures the relative perfusion deficit against simultaneously acquired adjacent healthy skin, thereby mitigating the influence of any short-lived hemodynamic response. Nevertheless, the degree of debridement was determined by clinical judgment rather than a standardized protocol, and pre- and postdebridement measurements were not compared in this study. Future studies should evaluate whether LSCI assessments conducted prior to debridement yield comparable discriminatory performance, which would further strengthen the applicability of this approach as a predebridement screening tool.

The detailed ROC analysis results, including the classification metrics, confusion matrix, and bootstrap-derived confidence intervals, are presented in Figure 3. Given the limited number of necrotic cases (*n* = 5), statistical uncertainty was assessed using patient-level bootstrap resampling (10,000 iterations), and the relatively wide confidence intervals reflect the inherent variability associated with small sample sizes. These intervals should be considered when the diagnostic metrics reported above are interpreted.

### Imaging efficiency and expanded clinical applicability

The ability to shorten the required imaging time without compromising diagnostic performance has important clinical implications. On the basis of the physiological rationale described in the STAR Methods section, we evaluated whether a 10-s acquisition could serve as a practical alternative to the conventional 60-s protocol. As shown in Figure 4A, the measurements observed using the 10-s and 60-s protocols demonstrated excellent agreement, with high correlation coefficients and minimal systematic biases in the Bland–Altman analysis. Both protocols yielded comparable diagnostic performance with respect to differentiating necrotic wounds from nonnecrotic wounds, with overlapping confidence intervals. The slightly higher classification accuracy observed with the 60-s protocol may be attributed to the averaging of transient perfusion fluctuations over multiple cardiac cycles. These findings support the feasibility of using the shortened 10-s protocol in clinical environments requiring rapid assessments, such as emergency care settings, or for patients who are unable to remain still for extended periods.

Nevertheless, the 95% limits of agreement (approximately ±20%) exceeded the predefined equivalence margin and were comparable in magnitude to the diagnostic cutoff of −22.6%. This finding indicates that for individual patients with normalized perfusion values near the decision boundary, the measurement variability between the two acquisition durations could, in principle, shift the classification outcome. In clinical practice, the 10-s protocol may therefore be most suitable as a rapid screening tool, with borderline cases confirmed by a full 60-s acquisition when the clinical situation permits.

**Figure 7:**
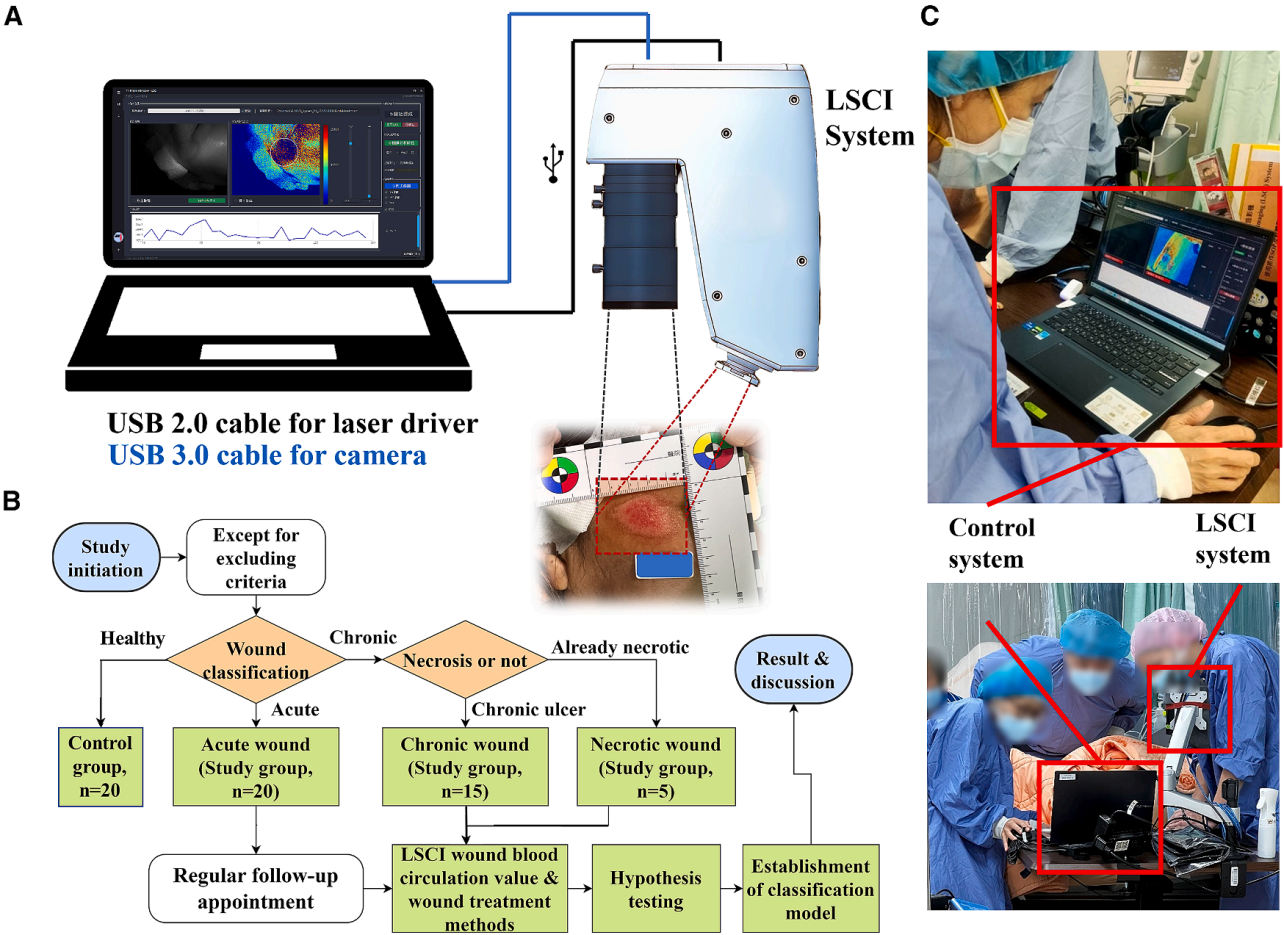
Process used to develop the architecture of the LSCI system and the wound healing evaluation model (A) Architecture of the LSCI system. The system comprises a laser light source module (an 805 nm NIR semiconductor laser diode with a circular pattern diffuser) and an image acquisition module (a CMOS sensor camera with a bandpass filter). (B) Experimental flowchart. A total of 60 participants were recruited, including 40 patients (20 with acute wounds, 15 with chronic ulcers, and 5 with necrotic wounds) and 20 participants without wound lesions. For each patient, adjacent healthy skin was simultaneously measured as a within-subject reference. Patients with acute wounds underwent follow-up measurements on days 0, 1, 2, and 5–15. (C) Clinical application demonstrates the noncontact wound perfusion measurement process implemented using the LSCI system.

Compared with previous studies focusing primarily on burn injuries using commercial systems such as the MoorLDI2-BI,^28^ the present study extended the application of LSCI to a broader spectrum of wound types, including abrasions, lacerations, and chronic ulcers. Furthermore, the compact and portable design of our custom LSCI system (Figure 7A), combined with its automated ROI tracking capabilities, enhanced measurement stability and can facilitate its integration into routine clinical workflows. Collectively, the findings in Figure 3 and Figure 4 demonstrate that LSCI offers both diagnostic accuracy and operational flexibility. The combination of perfusion-based necrosis discrimination and a shortened acquisition time positions LSCI as a promising tool for conducting noninvasive, real-time wound evaluations across diverse clinical environments.

### Noncontact and reduced assessment time for wound evaluation purposes

The increasing burden imposed by chronic wounds, combined with workforce shortages in wound care settings, underscores the need for efficient and objective assessment technologies.^29^^,^^30^^,^^31^

With the rapid advancement of smart technology, contactless blood circulation monitoring systems are emerging as promising tools for assessing clinical wounds. Previous research has demonstrated that LSCI can be used to assess the healing potential of burn wounds effectively.^28^ Building upon this foundation, the present study extends the clinical applicability of LSCI to a broader spectrum of wound types, including acute wounds, chronic ulcers, and necrotic wounds. Looking forward, the integration of LSCI into routine wound care workflows could enable the earlier identification of perfusion abnormalities, support objective clinical decision-making processes, and reduce the reliance on subjective visual assessments. The noncontact nature of LSCI is particularly advantageous in scenarios involving severe burns, extensive open wounds, or mass casualty events,^32^ where direct wound contact poses contamination risks and rapid triage of multiple patients is essential. In such situations, LSCI can provide real-time perfusion assessments to aid healthcare providers in making swift clinical decisions.

By using techniques such as LSCI, the time required for wound assessment can be shortened. For instance, in patients with limb burns, doctors traditionally require 1–2 days to determine wound condition. However, the application of LSCI systems can provide near real-time wound assessments within a short period, assisting medical teams in promptly devising effective treatment plans.^33^ The application of such contactless monitoring and assessment technologies will improve the quality of patient care, reduce the risks associated with chronic wounds, and lighten the workload imposed on healthcare workers.

Compared with traditional wound assessment methods, which rely on visual inspection and palpation, LSCI provides objective, quantitative microvascular data in a noncontact manner. Other emerging optical techniques, including hyperspectral imaging (HSI) and tissue SpO_2_ detection, have shown promise in wound evaluation scenarios^34^^,^^35^^,^^36^; however, their clinical adoption remains limited by high system complexity levels, high costs, and the need for standardized protocols. The LSCI approach adopted in this study offers practical advantages: rapid image acquisition (10–60 s), applicability across diverse wound types, and direct correlations with clinical outcomes. These characteristics position LSCI as a feasible adjunct for routine wound assessment purposes, particularly in time-sensitive or resource-limited settings. Notably, LSCI and HSI capture different physiological parameters: LSCI measures microvascular blood flow velocities, whereas HSI quantifies tissue oxygenation.^36^ Future multimodal approaches combining these techniques may therefore provide complementary information for implementing more comprehensive wound assessments.

Taken together, these findings indicate that LSCI can provide objective, near real-time perfusion measurements that help differentiate necrotic from nonnecrotic wounds across acute, chronic, and necrotic wound types, offering a rapid and quantitative complement to subjective visual inspection. The validated 10-s protocol retained comparable discriminatory performance while reducing wound exposure and patient discomfort, which is particularly valuable for elderly or immobile patients. Combined with its noncontact and real-time nature, these attributes position LSCI as a practical adjunct for wound assessment in time-constrained or resource-limited clinical settings.

### Future research directions

Several directions can be pursued in future work. First, expanding the sample size to include data from various wound types would increase the reliability of the statistical analyses. Longitudinal studies are needed to continuously monitor blood perfusion changes across different wound types to more accurately assess the dynamic healing process. The incorporation of other physiological indicators, such as SpO_2_ levels, temperature, and pH, could provide a more comprehensive assessment of the healing capacities of wounds and increase the accuracy of classification. Furthermore, optimizing statistical models and comparing different machine learning algorithms could lead to superior model selection results. The measurement environment requires further optimization, such as by using light-blocking devices and increasing the reproducibility of the measurement procedure. Future studies should also aim to further subdivide wound types, such as by distinguishing different degrees of necrosis and infection, to more precisely evaluate the changes in blood perfusion induced under various conditions. In addition to methodological refinements, future studies could explore the prognostic potential of LSCI. The present study was designed to discriminate wound types on the basis of single-time-point perfusion measurements, and the results demonstrated that necrotic wounds could be differentiated from nonnecrotic wounds with good accuracy (AUC = 0.88) when only the first measurement was used. The low perfusion observed in necrotic wounds may reflect long-standing regional vascular compromise in the affected area. Accordingly, wounds that eventually progress to necrosis may already exhibit reduced perfusion during the initial presentation before clinical signs of necrosis become apparent. Prospective studies could therefore investigate whether perfusion measurements obtained early during the development of wounds can predict which wounds are at risk for progression to necrosis, potentially extending the clinical utility of LSCI from wound discrimination to wound prognosis.

### Limitations of the study

This study had several limitations. The necrotic wound group included only 5 patients (*n* = 5), which limited statistical power. This limitation was primarily due to the clinical setting in which enrollment occurred, as most patients were recruited through the emergency department, where necrotic wounds are relatively less common. In clinical practice, patients with chronic or necrotic wounds are more frequently managed in other departments, such as plastic surgery or wound care units. Furthermore, the inherent complexity and urgency of necrotic wounds often require time-sensitive interventions, infection control, or cross-departmental coordination, all of which may further narrow the window for research enrollment even when eligible patients are present. The study was not powered to assess the influence or association of gender with perfusion outcomes, and no gender-based subgroup analysis was performed. In this study, only cross-sectional data were collected, limiting the number of long-term follow-up observations. The selection of the fourth examination (days 5–15) for cross-sectional comparison coincides with a phase of active angiogenesis in healing acute wounds, which may have amplified the perfusion contrast between the acute and necrotic groups compared with earlier examination time points. The agreement between the 10-s and 60-s protocols was evaluated exclusively using recordings from acute wound patients; whether the shortened acquisition yields comparable results in chronic or necrotic wounds, where perfusion levels and patient cooperation may differ, remains to be confirmed. LSCI technology can be affected by environmental factors such as ambient lighting and patient movements, potentially leading to measurement biases. In addition to environmental factors, the optical properties of tissues represent another potential source of measurement variability. Darker or more pigmented tissue absorbs more incident light, which could theoretically reduce the intensity of signals and affect speckle contrast calculations. In this study, the eschar and devitalized tissue were debrided prior to imaging to ensure that the measurements reflected the perfusion of the underlying viable tissue rather than optical surface artifacts. Furthermore, the speckle contrast formula (K = σ/⟨I⟩) was inherently normalized to determine the absolute intensity variations, and within-subject normalization against adjacent healthy-skin controls further accounted for individual differences among the baseline tissue properties. Nevertheless, residual optical heterogeneity may persist, and an independent validation conducted using complementary perfusion measurement techniques would further strengthen the reliability of the LSCI-based wound assessment process. In addition, the high prevalence of cardiovascular and cerebrovascular diseases in the necrotic group (60%) may have reduced the baseline perfusion of the adjacent healthy skin used as the normalization reference, potentially attenuating the observed normalized perfusion deficit in these patients. Although this study classified wounds into three categories (acute, chronic, and necrotic), the actual clinical presentations are often more complex^1^^,^^2^ and require further subclassification.^2^^,^^3^^,^^4^ The perfusion threshold values reported in this study are specific to our LSCI system configuration and are expressed in arbitrary units without absolute calibration to physiological blood flows. These values cannot be directly applied to other LSCI devices without system-specific validations, which represents a limitation that is common to all current LSCI implementations. Our sample population was recruited from a single medical center, which may not fully represent diverse patient populations across different healthcare settings.

## Resource availability

### Lead contact

Further information and requests for resources and reagents should be directed to and will be fulfilled by the lead contact, Lun-De Liao (ldliao@nhri.edu.tw).

### Materials availability

This study did not generate new unique reagents.

### Data and code availability

• All data reported in this paper, including the original LSC images, will be shared by the lead contact upon request.
• All original code will be shared by the lead contact upon reasonable request.
• Any additional information required to reanalyze the data reported in this paper is available from the lead contact, Lun-De Liao (ldliao@nhri.edu.tw), upon request.

## Acknowledgments

This research was supported in part by the 10.13039/100020595National Science and Technology Council of Taiwan through grant nos. NSTC 114-2221-E-400-001-MY3, NSTC 113-2221-E-400-003, NSTC 113-2218-E-007-026, 112-2221-E-005-042 and 111-2221-E-005-018; by the National Health Research Institutes of Taiwan through grant nos. BN-114-PP-15, BN-114-GP-02, BN-114-GP-03 and BN-114-GP-05; by the Ministry of Economic Affairs of Taiwan through grant nos. 114-EC-17-A-22-1906 and 114-EC-17-A22-1905; by the Metal Industries Research and Development Centre of Taiwan through grant no. K00069651K; and by the Southern Taiwan Science Park Bureau, National Science and Technology Council of Taiwan, through grant no. 113CH-1-03. Meng-Che Hsieh performed this research with funding support provided in part by the Doctoral Program in Tissue Engineering and Regenerative Medicine of 10.13039/501100004946National Chung Hsing University and the 10.13039/501100004737National Health Research Institutes.

## Author contributions

Conceptualization, M.C.H., L.D.L., Y.R.L., and P.Y.H.; methodology, M.C.H., L.D.L., Y.R.L., and P.Y.H.; resources, M.C.H., R.Z.L., and W.F.C.; investigation, Y.R.L. and P.Y.H.; formal analysis, M.C.H., Y.R.L., P.Y.H., R.Z.L., and W.F.C.; writing – original draft, M.C.H., R.Z.L., W.F.C., and L.D.L.; writing – review and editing, M.C.H., L.D.L., C.H.H., and C.T.S.C.

## Declaration of interests

The authors declare no competing interests.

## STAR★Methods

### Key resources table

**Table:** 

REAGENT or RESOURCE	SOURCE	IDENTIFIER
**Software and algorithms**		

MedCalc	MedCalc Software Ltd, Ostend, Belgium	https://www.medcalc.org/
Excel LTSC Professional Plus 2021	Microsoft, Seattle, Washington, USA	https://www.microsoft.com/zh-tw/microsoft-365
R (version 4.4.2)	R Foundation for Statistical Computing, Vienna, Austria	https://www.r-project.org/
LSCI program system	Hsieh et al.^24^	https://doi.org/10.1063/5.0172443

### Experimental model and study participant details

#### Setting and study population

This study was approved by the Human Experimentation Committee of Changhua Christian Hospital (IRB number: 211117), Taiwan. All participants provided written informed consent after receiving a full explanation of the study. Medical staff screened the patients who satisfied the imposed conditions for acute and chronic wounds, including chronic wounds such as diabetic foot ulcers, pressure sores, stasis ulcers, and ischemic ulcers. Acute wounds primarily involved nonhospitalized patients, including second-degree burns with body surface areas that were less than 15%, abrasions, and lacerations, with image assessments conducted on the 0th, 1st, 2nd, 5th, and (up to the) 15th days after injury. Patients with autoimmune diseases (e.g., systemic lupus erythematosus), organ failures, radiation ulcers, or malignant wounds were excluded for safety reasons. Once eligibility was confirmed, ROI data were collected via the use of noncontact LSCI. Participants with necrotic wounds underwent additional screening by clinicians to assess the extent of their necrosis, and patients requiring immediate surgical intervention at the time of screening were excluded. Necrosis was determined by attending emergency physicians on the basis of clinical examination, including the presence of tissue characterized by black or dark brown discoloration, eschar, slough, or devitalized tissue requiring debridement; this determination served as the reference standard for the subsequent diagnostic analysis. The patients who were confirmed to be eligible underwent a single LSCI assessment during their initial visit. Prior to imaging, the eschar and devitalized tissue were debrided by clinical personnel to ensure that the measurements reflected the perfusion of the underlying viable tissue within the wounds that were clinically diagnosed as necrotic. No follow-up assessments were conducted, as the clinical management of necrotic wounds often involves prompt treatment after the initial evaluation, which is not compatible with a standardized follow-up schedule. The patients with chronic wounds likewise underwent a single assessment during their initial visit. Although these patients attended regular outpatient appointments, their visit intervals were variable and typically ranged from weeks to months, precluding the collection of standardized longitudinal data; the measurements obtained at the first visit were therefore designated as the sole data points for this group. Written informed consent was obtained before the LSCI procedure, and participants were informed of their right to withdraw at any time. All imaging work was performed in a controlled, clean environment; the laser output and camera parameters were calibrated regularly, and the instruments were cleaned regularly. An adverse event reporting system alerted the principal investigator and IRB within 24 h of any erythema, worsening infection conditions, or other complications. If postimaging bleeding or infection deterioration occurred, hospital wound care, antibiotic therapy, or surgical referral protocols were activated to ensure prompt treatment.

Both male and female participants were enrolled in every group, and the gender distribution of each group is reported in Tables 1 and 2.

### Method details

#### LSCI and detection methods

In this study, we employed the laser speckle contrast imaging (LSCI) device developed by the NHRI Institute of Biomedical Engineering and Nanomedicine, Taiwan.^24^^,^^37^ The main structure of the device, as shown in Figure 7A, included a laser light source and an image acquisition system. We chose a near-infrared (NIR) semiconductor laser diode as the light source (wavelength: 805 nm; power: 500 mW; model: ML620G40; Thorlabs, USA) to enable light penetration through human skin and a semiconductor laser diode driver (model: SF8075-NM; Maiman Electronics, Russia) to regulate the output energy. We installed a circular pattern diffuser (model: ED1-C50; Thorlabs, USA) at the laser output, which caused the light to scatter at a 50° angle to achieve a uniform light energy distribution and increase the irradiated area. With respect to laser safety, the 805 nm semiconductor laser diode used in this system had a maximum output power of 500 mW (Class 3B); however, after passing through the diffuser and being measured at an operating distance of 30 cm, the power reaching the target surface was less than 0.52 mW, which is within the maximum permissible exposure (MPE) level stipulated by ANSI Z136.1–2022. At this working distance, the field of view was measured as approximately 20 × 15 cm, with an illumination power density ranging from 238.3 to 525.1 μW across the irradiated area, and 96.7% of the field of view fell within the full width at half maximum (FWHM), ensuring a uniform illumination distribution.^37^

The image acquisition system used an area scanning camera with a complementary metal oxide semiconductor (CMOS) sensor (model: acA1300-200μm; Basler, Germany) that captured monochrome images with a resolution of 1280 × 1024 pixels. Color photographs of the wound sites presented in this study were obtained separately by trained nursing staff using a standard digital camera at the time of each assessment, with a ruler placed adjacent to the wound to provide a dimensional reference; these images served as clinical documentation only and were not used during the perfusion quantification or statistical analysis processes. We installed a bandpass filter in front of the lens to filter out light sources with wavelengths other than 805 nm to ensure that the sensor received signals only from the NIR laser and that the captured images were generated by the laser.

The sensor transmitted the received data to a computer for processing, enabling visualization of blood flow in the irradiated area. The camera exposure time was set to 5 ms, with a frame acquisition rate of 15 FPS employed throughout all clinical measurements; these settings were selected to ensure adequate speckle modulation and a stable signal acquisition procedure under clinical ambient lighting conditions, which was consistent with the parameters validated in our prior work. Perfusion values (expressed in arbitrary units, AUs) were computed using spatial speckle contrast analysis, as detailed in our previously published validation study.^37^ Briefly, a 5×5-pixel sliding window was applied across each image frame to calculate the local speckle contrast level K (the ratio of the local standard deviation to the local mean intensity), and perfusion was represented as 1/K^2^, where higher perfusion values corresponded to lower speckle contrast levels. The mean perfusion value within the selected ROI was computed by spatially averaging 1/K^2^ across all pixels in the ROI for each frame and then temporally averaging the result across all frames within the 60-s recording. The spatial speckle contrast analysis technique was selected because it provides the optimal balance between spatial resolution and real-time output efficiency for our system hardware. At a working distance of 30 cm, the field of view (FoV) was approximately 20 × 15 cm.

An LSCI software system was used to track the target patient and calculate the perfusion of a broad area in real time. Users could adjust the light source and image sensor through the system, view real-time LSCI images, control the laser output, and select ROIs for recording and observing blood flow. The obtained data, including the NIR images, LSCI images, and blood flow.csv files, were stored.

The system supports two ROI selection approaches for accommodating different clinical scenarios. The first is contour-based ROI selection, in which clinical personnel manually trace the boundary of the ROI along the actual wound margin, providing complete coverage of the wound area; however, when patients exhibit unavoidable minor movements, the ROI may shift beyond the wound boundary and require reacquisition. To mitigate this issue, the system incorporates an automatic ROI tracking function on the basis of a channel and spatial reliability tracking algorithm,^24^ which compensates for positional drift in real time and substantially reduces the remeasurement rate, although the complete elimination of movement artifacts remains challenging. The second approach is fixed-shape ROI selection, in which a circular or custom small-area ROI is positioned at the center of the target wound away from the wound edge, ensuring that the ROI remains within the wound tissue without incorporating surrounding healthy skin; this approach is likewise combined with the automatic ROI tracking function.^24^ Because perfusion values are computed as the spatial mean of all pixels within the ROI, the measurement results are not substantially affected by the ROI size as long as healthy skin does not enter the selected area. The choice between the two approaches was made at the discretion of the clinical personnel on the basis of the wound characteristics and condition of each patient. Regardless of the selected approach, medical personnel confirmed that the ROI remained entirely within the wound area throughout each acquisition process, ensuring that the surrounding healthy skin was not incorporated into the perfusion measurement scheme.

#### Framework of the experimental procedure

A total of 60 participants were recruited for this study, comprising 40 patients in the study group (including 15 with chronic ulcers, 20 with acute wounds, and 5 with necrotic wounds) and 20 participants without wound lesions in the control group. The experimental workflow differed among wound types: acute wounds underwent longitudinal follow-up assessments, whereas chronic and necrotic wounds were evaluated using single-time-point measurements. The participants were grouped and processed according to the flowchart shown in Figure 7B. The clinical application process of LSCI technology is shown in Figure 7C, with medical personnel performing noncontact wound assessments. The LSCI system enables real-time perfusion measurements to be obtained while maintaining a safe distance from the wound site, eliminating the risk of contamination or additional trauma to fragile tissue. Patients with acute wounds regularly returned for scheduled follow-up visits, at which time LSCI measurements were performed on both the wound site and the adjacent healthy skin of the ipsilateral limb. The perfusion values at the wound site were compared with the simultaneously acquired adjacent healthy-skin measurements at each time point to assess the trajectory of the change in perfusion during the acute healing period. The perfusion values were normalized using adjacent healthy skin as a reference and expressed as percentage changes, which were calculated as (ROI_wound − ROI_healthy)/ROI_healthy × 100%.

Medical personnel activated the LSCI system, positioned each patient’s wound and adjacent healthy skin within the sensing area, and selected the ROIs to be measured. The adjacent healthy skin ROI was placed on the ipsilateral limb at an anatomical level comparable to that of the wound site, at a location free of any wound-related involvement, to serve as the perfusion reference. The distance between the LSCI camera and the wound surface was approximately 30 cm, and all the recordings were conducted under consistent ambient lighting conditions to maintain a stable speckle contrast level. Each measurement process lasted 60 s to determine the blood perfusion values of the ROI (AU), after which the applied wound treatment method was documented. This acquisition duration was selected because LSCI measurements may be influenced by pulsatile blood flows; averaging the acquired perfusion signals over a 60-s recording period reduces the instantaneous fluctuations that are associated with cardiac cycles and provides more stable tissue perfusion estimates. Considering that the resting heart rate in adults typically ranges from 60 to 100 beats per minute, a 60-s recording captures multiple complete cardiac cycles, ensuring that the averaged perfusion values reflect the underlying tissue states rather than transient hemodynamic variations. By the same reasoning, any arbitrary 10-s window within a static recording is expected to capture a physiologically representative sample, as it encompasses at least one complete cardiac cycle even at the lower bound of the normal resting heart rate. To evaluate the feasibility of a shorter acquisition protocol, a 10-s data subset was therefore additionally extracted by selecting the initial 10-s segment from each 60-s recording. The 10-s data were not acquired independently but rather derived from the same recording session, ensuring direct comparability between the two durations under identical physiological conditions. This design aimed to assess whether a 10-s acquisition period may provide a clinically practical alternative to the standard 60-s protocol in scenarios requiring rapid assessment without altering the measurement procedure.

These data were used for subsequent hypothesis testing work and for establishing classification models to verify whether significant perfusion value differences existed among the acute, chronic, and necrotic wounds, thereby providing a better understanding of the wound healing statuses. Finally, we analyzed and discussed the research results.

### Quantification and statistical analysis

For acute wound analysis, the within-subject normalized perfusion values were compared with 0 using the Wilcoxon signed-rank test. The corresponding *p* values were calculated on the basis of two-sided tests. The effect size is reported as *r*, which was calculated as *r* = |Z|/√N, where Z represents the standardized test statistic and N denotes the number of nonzero paired observations. The effect size *r* was interpreted as small (≥0.10), medium (≥0.30), or large (≥0.50) according to conventional criteria. Representative images illustrating tissue appearance and perfusion changes across examination times are presented in the results section.

We applied the Mann‒Whitney U test^38^^,^^39^^,^^40^ and the Kruskal‒Wallis test^41^ to verify whether significant differences existed among the mean perfusion values (AUs) for acute wounds, chronic ulcer wounds, and necrotic wounds; the results are presented in boxplots, and the hypothesis was tested by calculating *p* values. For acute wound analysis purposes, the normalized perfusion values (expressed as percent changes relative to the adjacent healthy skin) were compared with 0 using the Wilcoxon signed-rank test, and the resulting two-sided *p* value was reported for each comparison. For the Mann‒Whitney U test, the effect size *r* was calculated using the same formula (*r* = |Z|/√N) and interpretive benchmarks as described above, with N representing the total number of observations across both groups.^42^ Epsilon squared (*ε*^2^) was calculated as a nonparametric effect size index for the Kruskal–Wallis test. Values close to 0.01, 0.06, and 0.14 were interpreted as small, moderate, and large effects, respectively.^43^^,^^44^ The intraclass correlation coefficient (ICC) was computed using a two-way mixed-effects, absolute-agreement, single-rater model [ICC (3, 1)] along with 95% confidence intervals (CIs) to quantify the reliability of the two acquisition times.^45^ We plotted a scatter diagram of the perfusion values obtained at 10 s and 60 s, overlaid the identity line, and annotated it with the ICC (3, 1) and its CI. In parallel, Pearson’s correlation coefficient (*r*) and its *p* value were calculated to assess linear associations, and the corresponding regression line was added to the same plot, with the *r* and *p* value indicated. We applied the Bland–Altman equivalence analysis approach to assess the level of agreement between the 60- and 10-s ROI perfusion measurements and to evaluate whether short-duration imaging could substitute for the standard 60-s acquisition protocol. The relative percentage difference was used as the comparison metric. The results were interpreted using bias, the 95% CI, and the limit of agreement (LoA), with a predefined equivalence margin of ±10%. An ROC analysis was performed using the normalized perfusion values (%) to evaluate the ability of LSCI to differentiate necrotic wounds from nonnecrotic wounds. The diagnostic performance of the methods was assessed using the AUC, 95% CI, *p* value, and optimal cutoff determined by the Youden index.^27^ The optimal cutoff obtained from the ROC curve was used as a reference threshold for differentiating necrotic wounds from nonnecrotic wounds. In addition, the confusion matrix and precision, recall, and F1 score were calculated on the basis of the normalized perfusion values to provide a more comprehensive evaluation of the ability of the LSCI technique to identify necrotic wounds. The employed statistical analysis tools included the R language (version 4.4.2; R Foundation for Statistical Computing, Vienna, Austria), MedCalc Statistical Software (MedCalc 2023; Ostend, Belgium), and MS Excel (Excel LTSC Professional Plus 2021; Microsoft, Seattle, Washington, USA) for graphing and statistical calculation purposes. Owing to the small sample size of the necrotic group (*n* = 5), 95% confidence intervals for sensitivity, specificity, the PPV, the NPV, and the optimal cutoff were estimated using nonparametric bootstrap resampling (10,000 iterations) at the patient level. For each bootstrap sample, the ROC curve was recalculated, and the optimal cutoff was determined using the Youden index. Percentile-based 95% confidence intervals (2.5–97.5th percentiles) are reported.

#### Overview of the experimental dataset

The distribution of the sample groups and the corresponding statistical analyses conducted throughout this study are summarized in Table 5. The necrotic (*n* = 5) and chronic wound groups (*n* = 15) were analyzed via the Mann–Whitney U test and Kruskal–Wallis test to assess the differences in ROI perfusion values. Longitudinal comparisons within the acute wound group were performed using the Wilcoxon signed-rank test. However, because data were missing for one patient on day 2 (because of surgical debridement and the placement of artificial dermis) and because data were missing for another patient from days 5–15 (because that patient tested positive for COVID-19 and was placed under mandatory home isolation, precluding the scheduled clinic visit), only 19 samples were available at these time points. These data were analyzed to enable cross-sectional comparisons among necrotic, chronic, and acute wounds at later healing stages. Specifically, the fourth measurement (days 5–15) derived from the acute wound group was selected for cross-sectional comparison because this period represents an early but more stabilized phase of the healing process. At this stage, perfusion levels begin to reflect whether a wound is progressing toward recovery, making it a suitable reference point for comparison with chronic and necrotic wounds, which typically exhibit nonhealing perfusion profiles. In accordance with the clinical experience at the collaborating hospitals, this window also aligns with the time when treatment decisions are often adjusted on the basis of early healing responses. The control group (*n* = 20) was included in the healthy skin perfusion analyses. For diagnostic analysis purposes, ROC curve testing was performed using 39 participants, including 19 acute wounds, 15 chronic ulcers, and 5 necrotic wounds, to determine the optimal cutoff for identifying necrotic wounds on the basis of normalized perfusion values. A total of 78 perfusion recordings were collected from 20 patients with acute wounds through follow-up sessions to evaluate the feasibility of reducing the imaging time. These data were employed to perform time-based comparisons and a Bland–Altman equivalence analysis for assessing the agreement between the 60- and 10-s imaging durations. This structured dataset revealed the specific purpose of the study and the methodological application employed for each subgroup and supported the rigor of the subsequent statistical evaluation. To address the potential confounding effects of baseline characteristics, multivariable linear regression analyses were performed to evaluate whether the wound types were independently associated with the perfusion values. ROI perfusion (AU) was utilized as the dependent variable, and the wound type was included as a categorical independent variable, with the acute wound group used as the reference category. Age, mean arterial pressure (MAP), and diabetes status were included as covariates. Multicollinearity was assessed using variance inflation factors (VIFs). Statistical significance was defined as *p* < 0.05.
